# An Improved Whale Optimization Algorithm for the Clean Production Transformation of Automotive Body Painting

**DOI:** 10.3390/biomimetics10050273

**Published:** 2025-04-28

**Authors:** Qin Yang, Xinning Li, Teng Yang, Hu Wu, Liwen Zhang

**Affiliations:** 1 School of Mechanical Engineering, Shandong University of Technology, Zibo 255000, China; qyshang66@163.com (Q.Y.); zlw0623@163.com (L.Z.); 2 Graduate School, Gachon University, Seongnam-si 13120, Republic of Korea; 3 School of Intelligent Manufacturing, Zibo Polytechnic University, Zibo 255000, China; lxnwxg@126.com

**Keywords:** spraying, production, algorithm, optimization

## Abstract

Research on clean production in automotive painting processes is a core component of achieving green manufacturing, addressing environmental regulatory challenges, and advancing sustainable development in the automotive industry by reducing volatile organic compound (VOC) emissions, optimizing resource utilization, and minimizing energy consumption. To reduce pollutants generated by automotive painting processes and improve coating efficiency, this study proposes a clean production method for automotive body painting based on an improved whale optimization algorithm from the perspective of “low-carbon consumption and emission-reduced production”. A multi-level, multi-objective decision-making model is developed by integrating three dimensions of clean production: material flow (optimizing material costs), energy flow (minimizing painting energy consumption), and environmental emission flow (reducing carbon emissions and processing time). The whale optimization algorithm is enhanced through three key modifications: the incorporation of nonlinear convergence factors, elite opposition-based learning, and dynamic parameter self-adaptation, which are then applied to optimize the automotive painting model. Experimental validation using the painting processes of TJ Corporation’s New Energy Vehicles (NEVs) demonstrates the superiority of the proposed algorithm over the MHWOA, WOA-RBF, and WOA-VMD. Results show that the method achieves a 42.1% increase in coating production efficiency, over 98% exhaust gas purification rate, 18.2% average energy-saving improvement, and 17.9% reduction in manufacturing costs. This green transformation of low-carbon emission-reduction infrastructure in painting processes delivers significant economic and social benefits, positioning it as a sustainable solution for the automotive industry.

## 1. Introduction

The environmental problem is becoming more and more serious and has become a global problem with the rapid development of the global economy [1]. Cleaner production can improve the efficiency of resource utilization, reduce and avoid the generation of pollutants, protect and improve the environment, protect human health, and promote sustainable economic and social development [2]. The United Nations Environment Programme (UNEP) defines “cleaner production” as the continuous application of integrated preventive environmental strategies to production processes and products in order to reduce risks to humans and the environment [3]. Therefore, the essence of cleaner production is to emphasize three aspects: clean energy, a clean production process, and clean products, and to realize the “greening” of the whole process of production and the whole process of product life cycle. It is a new type of pollution prevention and control strategy proposed by the international community after summarizing the experience and lessons of industrial pollution control. It is of great significance as a way to prevent and control environmental pollution, reduce the burden of end treatment, improve the market competitiveness of enterprises, cope with the new situation of international trade, and implement the strategy for sustainable development [4].

It is necessary to integrate the concept of cleaner production (CP) into the production of enterprises for realizing the sustainable development of CP and to better adapt to the changing environment of social economy.

Yu et al. [5] integrated the concept of CP into all aspects of enterprise, including raw materials, waste, process control, process flow, product quality, staff quality, administrative management, etc., and established a cleaner production management model. Lv Lishu et al. [6] proposed a multi-level and multi-objective optimization model for the grinding process scheme for cleaner production in terms of energy saving and emission reduction in the grinding process. The experiment confirmed its effectiveness and put forward a corresponding energy saving and emission reduction strategy. Ma et al. [7] proposed a data-driven cleaner production strategy for energy-intensive manufacturing based on product life cycle management, and explained it through the life cycle of cleaner production data. Wang et al. [8] revealed the characteristics of changes in consumption structure forced by the transfer of low-carbon costs in enterprises, embedded the concept of low-carbon consumption in the utility function of consumers, established a production decision-making model of enterprises, and discussed the mechanism of the impact of the transfer of emission reduction costs on consumer demand changes. Sharma [9] studied the use of the best and worst method (BWM) and the Grey Decision Making Trial and Evaluation Laboratory (Grey DEMATEL) and grid framework to explore the transformation strategy and sub-strategy for the integration of cleaner production and environment, focusing on green, flexible, and inclusive development. The results show that strategies such as “using artificial intelligence and the Internet of Things for optimization (STG B)” and “combining block chain technology (STG D) (Incorporating Block chain technology)” are crucial. The study also classifies these strategies and their sub-strategies into causal groups, of which “using artificial intelligence and the Internet of Things for optimization (STG B) (Leveraging AI & IoT for optimization)” has the greatest impact. The adoption of advanced digital twins technology (STG C) (employing advanced digital twins technology) has the least impact. However, the implementation of cleaner production still faces many obstacles. Although the relevant policies for cleaner production have played a guiding and constraining role, they can help enterprises to achieve comprehensive utilization of resources and reduce pollutant emissions to a certain extent. However, cleaner production activities are a dynamic process. Technology is constantly improving. The latest market demand and resource management require enterprises to continuously improve their own management models. The existing industrial enterprise management system still rarely considers the sustainability of cleaner production and the lack of comprehensive consideration of resources, energy, economy, and environment [10].

With the increasingly serious energy and environmental problems, China has put forward the development concept of “innovation, coordination, green, open and sharing”. New energy vehicles and intelligent manufacturing in emerging industries have become the key strategies [11]. With the rapid development of the global automobile industry, the energy consumption, material consumption, and pollutant production of the whole automobile industry chain are also increasing sharply, especially for the coating process [12]. The coating production line is the production line with the largest investment and the highest process requirements in automobile production. It is also the process production line with the highest energy consumption and with three waste emissions. Hydropower consumption accounts for more than 60% [13] of the entire automobile production process, and the impact of a large number of pollutants on the environment is also increasingly serious. The wastewater discharged from the production process of coating pretreatment alone contains a large amount of pollutants such as acid, alkali, oil, heavy metals, nitrates, and phosphates. A large amount of electrophoresis wastewater from the electrophoresis primer process, due to its poor biodegradability, is currently widely used. With the chemical coagulation treatment method, it is difficult to meet the relevant pollutant discharge standards, and it will inevitably have serious adverse effects on the surrounding environment [14]. In the process of coating transportation, modulation, and coating, these organic solvents escape into the air and then produce a large amount of volatile organic compounds (VOCs), which not only pollute the atmospheric environment, but also directly cause great harm to human health [15]. Restricted by today’s environmental protection requirements, automobile coating production-related enterprises may be in trouble due to environmental pollution problems caused by the coating production process, which, in turn, limits the development of enterprises. In order to adapt to the development of the times, enterprises have carried out a lot of research on cleaner production to improve efficiency and the enterprise management level [16]. At present, there are different requirements for the cleaner production audit of automobile spraying production enterprises in different regions, and there are no uniform standards and requirements. The pass rate of the cleaner production audit is less than 40% [13]. Changing the concept; changing the traditional way of thinking; implementing cleaner production; accelerating technological innovation; reducing energy consumption, material consumption, and pollutants from the source; strengthening the control of the whole process of product production and the whole life cycle; and coordinating the development of economy and environment are needed for the sustainable development of enterprises. It is also the embodiment of corporate social responsibility and the inevitable trend towards developing a low-carbon economy.

Based on the moving rate of the tool center point (TCP) of the spraying robot, Wang [17] built the mathematical model of coating flow estimation. The spraying process plan of the robot and the body zoning were formulated through offline profiling simulation verification. Liu [18] further analyzed the basic principle of spraying copying technology. The influences of key parameters such as the spraying overlap rate, the robot spraying time, the TCP moving speed, and the coating flow rate on the paint film quality were determined, and the spraying process was optimized.

The vehicle scheduling problem in the painting process is expressed as a nonlinear 0–1 integer programming model, and the task optimization is carried out based on the genetic algorithm [19]. This indicates that the spraying problem for the vehicle can be optimized by the meta-heuristic algorithm. The whale optimization algorithm (WOA) is a swarm intelligence optimization algorithm based on the predatory behavior of humpback whales in nature. Proposed by Mirjalili and Lewis in 2016 [20], this algorithm simulates the “spiral bubble net hunting strategy” of humpback whales and has the characteristics of simple structure, few parameters, and easy implementation. It is widely used in fields such as function optimization, engineering optimization, and machine learning. In recent years, research relating to the WOA has mainly focused on algorithm improvement, multi-objective optimization, practical applications, and other aspects. The specific research situation is shown in the Table 1.

Based on this, the team studied the clean production management of automobile body spraying. On the basis of the combination of the value flow chart analysis technology of lean production theory and the ECRS optimization principle of process re-engineering, the artificial intelligence algorithm is further integrated to optimize the spraying process. The main innovations are as follows:

(1) An improved whale optimization algorithm (NEDWOA) is proposed, incorporating a nonlinear convergence factor, elite opposition-based learning, and adaptive dynamic parameters. The NEDWOA demonstrates significant improvements in global search capability, convergence speed, and robustness.
(2) The clean production problem in automotive body painting is defined as a multi-objective optimization issue involving material flow, energy flow, and environmental emissions in the painting process. From the perspective of “low-carbon consumption + emission-reducing production”, a multi-level, multi-objective decision-making model for automotive painting is established, considering both process and operational layers, with objectives including material cost, painting energy consumption, carbon emissions, and painting time.
(3) The NEDWOA is applied to optimize the automotive painting model, proposing a production method for automotive body painting based on the NEDWOA. Taking the new energy vehicles from the TJ Automotive Manufacturing Plant in China as an example, compared to other algorithms, the proposed algorithm achieves superior optimization results, improving painting production efficiency by 42.1%, achieving a waste gas purification rate of over 98%, enhancing average energy-saving effects by more than 18.2%, and reducing manufacturing costs by 17.9%.

The structure of this paper is as follows. Section 1 is the introduction, outlining the research background, current status, and significance of clean production, and emphasizing the urgency of clean production management in the automotive painting industry. Section 2 presents the improved whale optimization algorithm (NEDWOA). Section 3 details the multi-level, multi-objective optimization method for the painting process based on the NEDWOA. Section 4 conducts experimental validation before and after optimization, which is followed by result analysis. The Section 5 summarizes the research conducted in this paper.

## 2. Improved Whale Optimization Algorithm

The whale optimization algorithm (WOA) is a swarm intelligence optimization method inspired by the bubble-net hunting behavior of humpback whales, and was first proposed by Mirjalili and Lewis [20]. This algorithm mimics the “spiral bubble-net feeding strategy” of whales, and is characterized by structural simplicity, minimal parameter requirements, and ease of implementation. It has been widely applied to function optimization, engineering design, and machine learning. Recent research on the WOA has focused on three areas: algorithmic enhancements, multi-objective optimization, and practical industrial applications. To address the WOA’s issues of easily falling into local optima and slow convergence speed, researchers have proposed various improvement strategies, such as introducing nonlinear convergence factors [29], adaptive weights [44], and chaotic mapping. Hybrid approaches combining the WOA with other optimization algorithms (e.g., Particle Swarm Optimization [PSO] and the genetic algorithm [GA]) have also been developed to enhance global search capability and robustness. For multi-objective optimization, extensions like the Pareto front-based multi-objective WOA (MOWOA) have been explored.

To further improve the WOA’s convergence and robustness, this study introduces three enhancement strategies:

(1) Nonlinear convergence factor: replaces the linear-decreasing convergence factor with a nonlinear version to flexibly balance exploration and exploitation.
(2) Elite opposition-based learning: generates reverse solutions from high-quality elite individuals to increase population diversity.
(3) Dynamic parameter self-adaptation: adjusts parameters in real time based on search states to enhance adaptability.

The improved algorithm, named NEDWOA (nonlinear convergence factor + elite opposition-based learning + dynamic parameter adaptation), implements these strategies as follows.

### 2.1. Improved Strategy

(1) Nonlinear Convergence Factor

In the original WOA, the convergence factor, a, decreases linearly, resulting in rigid search step size adjustments that cannot adapt to complex optimization processes. The proposed exponential nonlinear convergence factor is defined as

(1) $$a(t)=a_{init}\cdot 1-{\frac{t}{T_{\max}}}^{\gamma }$$

where the hyperparameter controls the decay rate *γ* > 1 (typically γ = 2). The initial convergence factor *α_init_* is 2.

In the early phase (*t* << T_max_), rapid decay of α enables larger step sizes, which can accelerate global exploration. In the late phase (*t* → T_max_), gradual decay of α refines local exploitation, stabilizing convergence toward the optimal solution.

This exponential formulation allows dynamic adaptation to problem complexity and scale, significantly enhancing the algorithm’s flexibility compared to the linear counterpart.

(2) Elite Opposition-Based Learning

To address the premature convergence caused by decaying population diversity, this study employs elite opposition-based learning to expand search space coverage. For each generation, the top k fitness-ranked elite individuals are selected, and their opposition solutions are generated and selectively retained.

For an elite individual X_elite_, its opposition solution is computed as

*X*_oppo_ = UB + LB − *X*_elite_ (2)

where UB and LB represent the variable’s upper and lower bounds, respectively. The superior solutions between X_elite_ and X_oppo_ are retained.

The opposition solutions are integrated into the population while maintaining a constant total population size by removing the worst-performing individuals. This strategy effectively mitigates local optima stagnation and demonstrates enhanced performance in multimodal optimization problems due to its systematic exploration–exploitation balance.

(3) Dynamic Parameter Self-Adaptation

To resolve the limitations of fixed parameters in the original algorithm, which struggles to adapt to distinct optimization phases, this study introduces a fitness variance-driven dynamic parameter adjustment mechanism. The fitness variance calculation is written as

(3) $$\sigma^{2}(t)={\frac{1}{N}}_{i=1}^{N}f(X_{i})-{\bar{f}}^{2}$$

where $\bar{f}$ is the average fitness of the current population and *N* is population size.

Dynamic adjustment rules are as follows. When σ^2^(t) is small, low fitness variance indicates population convergence. Increasing the spiral coefficient *b* intensifies local exploitation.

(4) $$b(t)=b_{\min}+(b_{\max}-b_{\min})\cdot \frac{\sigma^{2}(t)}{\sigma_{\max}^{2}}$$

When σ^2^(t) is large, high fitness variance indicates population diversity. Increasing the random search probability *p* strengthens global exploration.

(5) $$p(t)=p_{min}+(p_{max}-p_{min})\cdot (1-\frac{\sigma^{2}(t)}{\sigma_{max}^{2}})$$

This strategy enables parameters to self-adapt based on population states, balances exploration and exploitation, and enhances the algorithm’s adaptability to complex optimization problems.

### 2.2. NEDWOA

The optimization strategy of the NEDWOA consists of three stages: encircling prey, spiral bubble net predation, and global exploration. The action equation of encircling prey is expressed as

(6) $$X_{n(t+1)}^{\prime }=\sigma^{2}(t)\cdot X_{t}^{\prime \Delta }-A\cdot D$$

where $X_{t}^{\prime \Delta }=(X_{1}^{\prime \Delta },X_{2}^{\prime \Delta },\ldots ,X_{d}^{\prime \Delta })$ is the optimal prey position. Whales initially choose random individual positions as search targets. The optimal position is gradually updated and replaced with the increasing iterations. A is the coefficient vector. D is the distance between the current individual and the current optimal individual. A and D can be written as:

(7) $$A=2a\cdot r_{rand}-a$$

(8) $$D=\left|2r_{rand}\cdot X_{t}^{\Delta }-X_{t}\right|$$

where a is the convergence factor (Equation (1)) and ***r***_rand_ is a random vector from 0 to 1.

The action equation of spiral bubble net predation is expressed as

(9) $$X_{n(t+1)}^{\prime }=\sigma^{2}(t)\cdot X_{t}^{\prime \Delta }+D^{\prime }\cdot e^{bl}\cdot \cos(2\pi l)$$

where b is the spiral coefficient (Equation (4)) and *l* is the random number between [−1, 1].

Both of the above two belong to the development stage of the algorithm. The selection probability *p* (Equation (5)) of each of the two stages accounts for 50%. The mathematical models of development stage can be written as follows:

(10) $$X_{n(t+1)}^{\prime }=\sigma^{2}(t)\cdot X_{t}^{\prime \Delta }-A\cdot D,\begin{array}{cc} & p<0.5 \end{array}$$

(11) $$X_{n(t+1)}^{\prime }=\sigma^{2}(t)\cdot X_{t}^{\prime \Delta }+D^{\prime }\cdot e^{bl}\cdot \cos(2\pi l),p\geq 0.5$$

The action equation of global exploration phase is expressed as

(12) $$X_{n(t+1)}^{\prime }=\sigma^{2}(t)\cdot X_{t}^{r}-A\cdot D^{\prime \prime }$$

where $X_{t}^{r}$ is the randomly selected location of the individual whale. D″ is the distance between the current individual ***X****_it_* and the random individual $X_{t}^{r}$ in the *t*th iteration, which is written as

(13) $$D^{\prime \prime }=\left|2r_{rand}\cdot X_{t}^{r}-X_{nt}\right|$$

The choice of the above three phases depends on the parameters *p* and A. The spiral bubble-net phase is selected when *p* ≥ 0.5. Encircling prey is selected when *p* < 0.5 and |A| < 1. The global exploration phase is selected when *p* < 0.5 and |A| ≥ 1 to search for a more optimal solution. The pseudo-code of the NEDWOA is described in Algorithm 1.
**Algorithm 1**: Pseudo-code of the NEDWOA**Input:** number of search agents: N, Dim, and Max_Iter **Output**: optimal fitness value Initialize position of every individual whale Calculate the fitness value for all search agents The search agent with the best fitness was selected as the lead whale While lter < Max_Iter Calculating the nonlinear convergence factor a by the Equation (1) Calculating the dynamic parameter self-adaptation *σ*^2^(*t*) by the Equation (3) Calculating the spiral coefficient b by the Equation (4) Calculating the random search probability p by the Equation (5) Calculating the coefficient vector **A** by the Equation (7) Calculating the coefficient vector **D** by the Equation (8) Assign random numbers between [−1, 1] to l and between [0, 1] to r_rand_, respectively, If (*p* < 0.5)     If (|A| < 1)     Update the position of the current search agent by the Equation (6)     else if (|A| ≥ 1)     Select a random search agent X_rand_     Update the position of the current search agent by the Equation (12)     End if else if (*p* ≥ 0.5) Update the position of the current search by the Equation (11) End if End for Update the current position of whale populations using elite opposition-based learning by the Equation (2) Update a, σ, l, b, and p Check if the agent is out of the search boundary and correct it Calculate the fitness value for all search agents Sort populations in ascending order according to fitness The search agents are sorted in ascending order according to the fitness value Update X* if there is a better solution Iter = Iter + 1 End while Return X* and optimal fitness value

### 2.3. Experimental Results and Discussion

(1) Benchmark functions

The 23 benchmark functions shown in Table 2 were selected to verify the performance of the proposed NEDWOA.

(2) Effect of each proposed component

Table 3 shows the comparison results for the kinds of improved strategies. WOAa is only improved by nonlinear convergence factor Equation (1). WOAo is only improved by elite opposition-based learning Equation (2). WOAσ is only improved by dynamic parameter self-adaptation $\sigma^{2}(t)$ Equation (3). All experiments were carried out in the environment of Intel(R) Xeon(R) W-2255, 64 GB RAM, and MATLAB (2021a). Set the population size as 30 and the maximum iteration times as 500. The parameters of WOA are set as b = 1, c = 2·*rand*, ***a*** = 2 − 2*t*/*t*_max_, and ***A*** = (c − 1)·***a***. The parameters of related NEDWOA are set as bϵ [1, 3], pϵ [0.1, 0.5]. For each test function, all algorithms in the same dimension run independently for 30 times. The average values (Avg) were used to test the convergence accuracy of the algorithm. The standard deviation (Std) was used to test the stability.

Then, NEDWOA was compared with the WOA and its related variants WOA-RBF [45], WOA-VMD [46], and MSWOA [39]. The optimization results are shown in Table 4. The NEDWOA can obtain the best values.

## 3. Analysis of “Low Carbon Consumption + Emission Reduction Production” Spraying Model

### 3.1. Analysis of Automobile Spraying Process Problems

**Figure 1:**
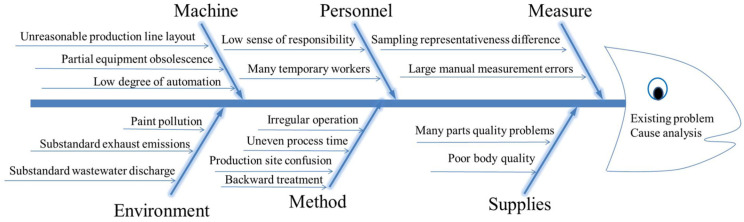
Cause analysis of body painting production process fishbone diagram.

The team conducted a survey of a number of automobile companies. By studying the causal relationship between the body painting production process problems, the specific reasons were analyzed using the fishbone diagram method, as shown in Figure 1. By using the fishbone diagram to analyze the existing problems of low coating efficiency, high production cost, substandard environmental protection, and inadequate on-site management, the causes of the problems were found: from the personnel aspect, there was a low sense of responsibility and many temporary workers, and from the machine aspect, there were some problems such as unreasonable layout of production line, obsolete equipment, and a low degree of automation. From the materials aspect, there were many problems in terms of the quality of accessories and the poor quality of the body, and there were some problems in terms of the method, such as non-standard operation, unbalanced process time, chaotic production site, and backward management methods. From the environmental aspect, there were problems such as a large amount of paint pollution, substandard exhaust gas emissions, and substandard wastewater emissions, and there are some problems in terms of measurement, such as poor sampling representativeness and large manual measurement errors.

**Figure 2:**
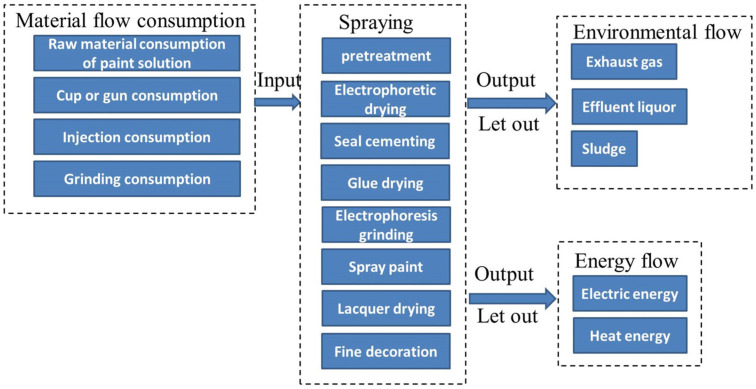
“Three-stream” analysis of spray-painting process.

Cleaner production is a management method used to prevent the whole production process of products. The input for the whole spraying process includes raw materials, auxiliary materials, and energy sources such as electricity, natural gas, and coal. The output includes energy sources such as electric energy and heat energy, and emissions such as gas, waste, and waste liquid. Therefore, the spraying model can be established from three perspectives: material flow consumption M, energy flow consumption E, and environmental emission flow consumption T. Material flow consumption includes the consumption of various materials, m, in the spraying process, such as water sandpaper, primer, topcoat, putty, oil ash, etc. Energy flow consumption, E, includes the consumption of all kinds of energy, e, and environmental emission flow includes the emission of various pollutants, t, such as paint sludge, waste gas, and waste liquid. The input and output analysis of the spraying process is shown in Figure 2.

### 3.2. Energy Consumption Model

From the perspective of the spraying state and energy flow, the energy consumption of the spraying process can be decomposed into two modules, including energy consumption E_spray_ and other energy consumption E_others_ (energy consumption E_start_ in start-up stage, energy consumption E_await_ in standby stage, and energy consumption E_close_ in shutdown stage), as shown in Figure 2.

The energy consumption in the shutdown stage is an instantaneous mutation of energy, which is generally not considered. The total energy consumption of the spraying process can be expressed as [6]:

(14) $$\begin{array}{cc} E_{total} & =E_{spray}{+E}_{\mathrm{others}}=E_{spray}+E_{\mathrm{start}}+E_{\mathrm{await}}\\ & =\int_{0}^{t_{spray}}P_{spray}\left(t\right)dt+\int_{0}^{t_{start}}P_{start}\left(t\right)dt+\int_{0}^{t_{await}}P_{\mathrm{await}}\left(t\right)dt \end{array}$$

where *P_spray_*, *P_start_*, and *P_await_* are the power of painting stage, start-up stage, and standby stage, respectively. *t_spray_*, *t_start_*, and *t_await_* are the time of the painting stage, start-up stage, and standby stage, respectively.

#### 3.2.1. Energy Consumption in the Painting Stage

The energy consumption generated in the painting stage is the most important energy consumption in the spraying process. It mainly refers to the energy consumed by the painting equipment to spray the paint onto the car body under a certain air pressure. The energy consumption in the spray-painting stage mainly includes the energy consumption generated by the collision power, *Pe*, between the paint droplets, the additional load loss power, *P_loss_*, and the energy consumption, *W*, generated by the collision between the paint and the car body, which can be expressed as:

(15) $$E_{spray}=\int_{0}^{t_{spray}}P_{spray}\left(t\right)dt+W=\int_{0}^{t_{spray}}\left(P_{e}\left(t\right)+P_{loss}\left(t\right)\right)dt+W$$

The power generated by the paint spray is related to the force generated, including the normal force and tangential force generated when the paint is in contact with the body. The paint fluid is considered as an aggregate of a large number of paint droplets. The paint droplets are regarded as discrete elements. Assuming that they maintain a spherical structure when colliding with the body, the flow of the paint droplets can be described based on Newton’s second law [46]:

(16) $$m_{i}\frac{d\nu_{i}}{dt}=\sum_{j=1,i\neq j}^{N}F_{ij}+m_{i}g$$

(17) $$I_{i}\frac{d\omega_{i}}{dt}=\sum_{j=1,i\neq j}^{N}T_{ij}$$

where *m*_i_ represents the mass of paint droplet, *i*; *v*_i_ and ω_i_ are the linear velocity and angular velocity of paint droplet *i*, respectively; *F_ij_* represents the contact force of paint droplet *i* from discrete paint droplet *j*; *I_i_* is the moment of inertia of paint droplet *i*; and *T_ij_* is the torque generated by the tangential component of the contact force between paint droplets.

Considering the adhesion of the contact surface between the paint droplets, this paper uses the Johnson–Kendall–Roberts (JKR) contact theory [46,47] to calculate the normal contact force between the paint droplets. Using the energy representation method, the contact surface energy, Δ, formed by the unit contact area of the two spherical particles is used to simulate the adhesion of two droplet particles with a contact radius of *r*. Then the collision normal force, *F_n_*, between the two droplets can be expressed as:

(18) Fn=−4πΔE∗r32+4E∗3R∗r3−256lneln2e+π22m∗E∗(R∗λn)0.5vnrel

The collision normal force, Ft, between two droplets can be expressed as:

(19) $$F_{t}=\min\left[\mu_{s}F_{n},\left|-8E^{*}\sqrt{R^{*}\lambda_{n}}\lambda_{t}-2\sqrt{\frac{5}{6}}\frac{\ln e}{\sqrt{\ln^{2}e+\pi^{2}}}\sqrt{8m^{*}E^{*}{\left(R^{*}\lambda_{n}\right)}^{0.5}}v_{trel}\right|\right]$$

where $\lambda_{n}=\frac{r^{2}}{R^{*}}-\sqrt{\frac{4\pi \Delta r}{E^{*}}}$, $r=\sqrt[{3}]{\frac{9\pi R^{*2}\Delta }{2E^{*}}}$, and $E^{*}$ are the equivalent elastic modulus of droplets as a function of Young’s modulus *E* and Poisson ratio *v*, $E^{*}={\left(\frac{1-v_{i}^{2}}{E_{i}}+\frac{1-v_{j}^{2}}{E_{j}}\right)}^{-1}$; $R^{*}$ is the equivalent droplet radius based on the droplet radius R, $R^{*}={\left(\frac{1}{R_{i}}+\frac{1}{R_{j}}\right)}^{-1}$; $m^{*}$ is the equivalent mass, $m^{*}={\left(\frac{1}{m_{i}}+\frac{1}{m_{j}}\right)}^{-1}$; e is the restitution coefficient of droplet collision; and $\mu_{s}$ is the static friction coefficient between droplets. $v_{nrel}$ and $v_{trel}$ are the normal relative velocity and tangential relative velocity between two droplets, respectively.

The loss power *P_e_* of paint droplet collision is:

(20) $$P_{e}=\sqrt{F_{n}^{2}+F_{t}^{2}}vN$$

where *v* is the linear velocity of the paint and N is the total number of paint particles in the spray arc zone.

The energy consumption of paint droplets impacting on automobile body is related to the density of working fluid, the velocity *v*_0_ of droplets impacting on solid surface, the initial diameter *D*_0_ of droplets, the surface tension coefficient σ of droplets, and the dynamic viscosity *μ* of droplets. The initial kinetic energy before impact [48] is:

(21) $$KE_{b}=(\frac{1}{2}\rho v_{0}^{2})(\frac{\pi }{6}D_{0}^{3})$$

Surface energy before impact can be written as:

(22) $$SE_{b}=\pi D_{0}^{2}\sigma$$

After the impact, the droplet reaches maximum spreading, and the instantaneous kinetic energy *KE_a_* = 0. The surface energy after impact is:

(23) $$SE_{a}=\frac{\pi D_{\max}^{2}\sigma }{4}(1-\cos\theta_{A})$$

where *θ_A_* is the advancing contact angle.

According to the law of conservation of energy, *KE_b_* + *SE_b_* = *KE_a_* + *Se_a_* + *W*, the work, *W*, to overcome the viscous force is [49]:

(24) $$W=\frac{\pi }{3}\rho v_{0}^{2}D_{0}D_{\max}^{2}\sqrt{\frac{\mu }{\rho v_{0}D_{0}}}$$

It is generally believed that the additional load loss power is (0.1~0.2) times that of the normal work loss power. Therefore, the energy consumption generated by the additional load is set as $0.2\times \left(\int_{0}^{t_{spray}}P_{e}\left(t\right)dt+W\right)$.

#### 3.2.2. Other Energy Consumption

Other energy consumption includes start-up phase and standby phase energy consumption. Energy consumption in the start-up phase, *E_start_*, is the energy needed to maintain the stable operation of the spraying robot after opening, which is related to the basic start-up power and start-up time of the robot. The basic start-up power is composed of the control system power, *P_c_*, and the pneumatic system power, *P_p_*, and the start-up time is determined by the robot’s own characteristics and procedures. The standby phase refers to the energy consumed by all other auxiliary systems after the spraying robot is turned on, including the energy consumption of the cooling device system, *E_cool_*, the energy consumption of the air-drying device system, *E_dry_*, the lighting device system, *E_light_*, and the temperature control system energy consumption, *E_tem_*. Other energy consumption based on this spraying process can be summarized as:

(25) $$\begin{array}{cc} E_{others} & =E_{start}+E_{await}=P_{start}t_{start}+P_{await}t_{await}\\ & =\int_{0}^{t_{start}}\left(P_{c}\left(t\right)+P_{p}\left(t\right)\right)dt+\int_{0}^{t_{await}}\left(P_{c}\left(t\right)+P_{p}\left(t\right)\right)dt+\sum_{k=0}^{K}v\left(k\right)\int_{0}^{t_{k}}P_{k}\left(t\right)dt\\ & =\int_{0}^{t_{start}+t_{await}}\left(P_{c}\left(t\right)+P_{p}\left(t\right)\right)dt+\sum_{k=0}^{K}v\left(k\right)\int_{0}^{t_{k}}P_{k}\left(t\right)dt \end{array}$$

where *v*(*k*) is the working state of an auxiliary system, and *v*(*k*) = 0 or 1 represents the stop and run states, respectively. *P_k_* is the stable operation power of an auxiliary system.

### 3.3. Carbon Emissions Model

The total carbon emission of the spraying process can be expressed as:

(26) $$C_{total}=C\left(M,E,T\right)=C_{m}+C_{e}+C_{t}=\sum_{n=1}^{n_{0}}M_{n}f_{m}+\sum_{g=1}^{g_{0}}E_{g}f_{e}+\sum_{s=1}^{s_{0}}T_{s}f_{t}$$

where *C_m_*, *C_e_*, and *C_t_* are carbon emissions from material flow, energy flow, and environmental emission flow, respectively, and *f_m_*, *f_e_*, and *f_t_* are carbon emission factors from material flow, energy flow, and environmental emission flow, respectively [50].

The carbon emission of the material flow mainly refers to the carbon emissions from the material consumed during the operation of the spraying system during its preparation process. The solvents used in paint and coating formulations are the main source of carbon emissions. Carbon emissions can be calculated using the following equation:

(27) $$C_{m}=m_{p}f_{mp}$$

where $m_{p}$ is the mass of paint or coating and $f_{mp}$ is the carbon emission factor of the material.

A large amount of electric energy will be consumed in the process of automobile body spraying. The use of electric energy itself does not produce carbon emissions. Here, the carbon emissions generated during the use of electric energy are calculated; that is:

(28) $$C_{e}=E_{total}f_{e}$$

The emissions that cause environmental impacts during the coating process include emissions into air, water (excessive paint spraying), and soil (paint sludge) [51]. Therefore, the carbon emissions generated by the environmental emission flow include carbon emissions $C_{t}^{ps}$ from waste treatment such as paint sludge, carbon emissions $C_{t}^{pl}$ from waste liquid treatment, and carbon emissions $C_{t}^{pg}$ from waste gas. The calculation formula is as follows:

(29) $$C_{t}=C_{t}^{ps}+C_{t}^{pl}+C_{t}^{pg}=m_{ws}f_{t}^{ws}+\rho_{wl}V_{wl}f_{t}^{wl}+\frac{M_{g}}{V_{m}}V_{wg}f_{t}^{wg}$$

where *m_ws_* is the mass of solid waste, *ρ_wl_* is the density of waste liquid, *V_vl_* is the discharge volume of waste liquid, Mg is the molar mass of waste gas, *V_m_* is the molar volume of waste gas, *V_wg_* is the volume of waste gas, and $f_{t}^{ws}$, $f_{t}^{wl}$, and $f_{t}^{wg}$ are the carbon emission factors for waste, waste liquid, and waste gas treatment, respectively.

## 4. Multi-Layer and Multi-Objective Optimization Model and Method for Spraying Process

### 4.1. Objective Function

The core of clean production management in the automobile body spraying process is to reduce energy consumption and promote green environmental protection, along with reducing costs and improving efficiency as much as possible. In view of the problems existing in automobile spraying, the spraying process flow is optimized and the real-time process re-engineering is carried out. Therefore, the optimization process for the automobile body spraying process scheme is a multi-objective optimization problem.

In this paper, spraying energy consumption, spraying carbon emission, and spraying efficiency are taken as optimization objective functions, and the combinatorial optimization problem of the reasonable selection of process layer (spraying method, spraying gun, body, and other spraying elements) and process layer (spraying process parameters) is studied. That is, the simultaneous optimization method for energy consumption, carbon emission, and spraying time under the generalized general mathematical model. The optimization objective function for low energy consumption, low carbon, and high efficiency manufacturing can be expressed as

(30) $$\min f\left(x\right)=y\left(E_{T},C_{T},T_{T}\right)$$

The energy consumption of the entire spraying process route should take into account the energy consumption generated by each spraying process, *i* (*i* = 1, 2, …, *i*_0_), which can be expressed as

(31) $$E_{T}=\sum_{i=1}^{i_{0}}E_{total}$$

The carbon emissions generated by the entire spraying process are similar to the energy consumption and can be expressed as

(32) $$C_{T}=\sum_{i=1}^{i_{0}}C_{total}$$

The high efficiency of the spraying process route is reflected in the shortest time of the spraying process. The total spraying time, *T_T_*, includes the body spraying time, the paint adjustment time, and the paint replacement time.

The body spraying time, *t_p_*, is determined by the spraying path length, *L*, and the robot spraying feed speed, *v_p_*, which can be written as

(33) $$t_{p}=\frac{L}{v_{p}}$$

In the painting process, if the two adjacent processes use different paint liquid, the paint liquid needs to be replaced. The paint liquid replacement time, *t_pc_*, in the painting process route can be obtained by the sum of the paint liquid replacement time of each process.

(34) $$\begin{array}{l} t_{pc}=t_{opc}\sum_{i=1}^{i_{0}}\left(W_{i+1}^{\prime }-W_{i}^{\prime }\right)\\ \left(W_{i+1}^{\prime }-W_{i}^{\prime }\right)=\left\{\begin{array}{c} \begin{array}{cc} 1 & W_{i+1}^{\prime }\neq W_{i}^{\prime } \end{array}\\ \begin{array}{cc} 0 & W_{i+1}^{\prime }=W_{i}^{\prime } \end{array} \end{array}\right. \end{array}$$

where *t_opc_* is the single paint replacement time and $W_{i}^{\prime }$ is the paint number used in the i-th spraying process. If one paint is sprayed in two adjacent processes, $W_{i+1}^{\prime }-W_{i}^{\prime }$ = 0, otherwise $W_{i+1}^{\prime }-W_{i}^{\prime }$ = 1.

### 4.2. Constraint Condition

The constraints of the process layer and the process layer are discussed, respectively, in this part. The spraying process for automobile bodies can be divided into rationality constraint and optimal constraint according to its mandatory difference at the process layer. The optimal solution for the process route optimization must meet the rationality constraints and the optimal constraints as far as possible.

The reasonable constraints include: ① the normal process flow is unchanged. Firstly, the pretreatment is carried out, and then the spray painting is carried out after electrophoresis drying, gel injection, and electrophoresis grinding; ② first, give priority to ensure the quality of work in the main process (first coarse and then fine, advanced coarse sealing glue injection repair glue and then fine sealing glue injection repair glue) and for grinding, also abide by the principle of first coarse and then fine; ③ it is ensured that the follow-up process does not destroy the characteristics of the previous process; and ④ the general constraints should also meet the principle of non-destructive constraints. Optimal constraints usually consider the constraints generated for the optimization objectives (including high efficiency, low cost, high precision, etc.) and the principles of improving the processing efficiency and economy of replacing machines, tools, and equipment.

The mathematical model of the constraint process is as follows:

(35) $$\begin{array}{l} RC_{i}\left(x\right)=0\begin{array}{cc} & i=1,2,\ldots ,i_{0} \end{array}\\ OC_{i}\left(x\right)=0\begin{array}{cc} & i=1,2,\ldots ,i_{0} \end{array}\\ x\in \Omega \begin{array}{cc} & \Omega =x_{1},x_{2},\ldots ,x_{i0} \end{array} \end{array}$$

where $RC_{i}\left(x\right)$ and $OC_{i}\left(x\right)$ are the rationality constraint and the optimization constraint, respectively, Ω is all the solutions in the spraying element, and there is i0! Due to the existence of constraints, the actual feasible spraying process route is less than i0!

It is necessary to detect the spraying path of the spraying robot when the spraying robot operates according to the planned spraying path to prevent the collision between the body and the spray gun at the process layer, that is, any point, *p* (*x_p_*, *y_p_*, and *z_p_*), on the spraying path of the robot meets the following conditions:

(36) $$\begin{array}{l} {\left(x_{p}-x_{pc}\right)}^{2}+{\left(y_{p}-y_{pc}\right)}^{2}+{\left(z_{p}-z_{pc}\right)}^{2}\geq D_{safe}^{2}\\ D_{\min}\geq D_{safe} \end{array}$$

where *x_pc_*, *y_pc_*, and *z_pc_* represent the three-dimensional coordinates of the point cloud contour data set, respectively, *D_min_* is the shortest distance between *p* and the data set, and *D_safe_* is the safe distance without collision.

During the spraying process, the air flow rate changes too much, which may cause the air control unit (ACU) to fail to respond, and the film thickness is randomly thicker. Spraying parameters, such as air flow, coating flow, and voltage, should avoid significant changes. Constraints are added to the kinematics and dynamics of each joint of the spraying robot manipulator. The kinematic constraints are written as follows:

(37) $$\left\{\begin{array}{l} \left|{\dot{\theta }}^{s}(t)\right|\leq V_{\max}^{s}\\ \left|{\ddot{\theta }}^{s}(t)\right|\leq A_{\max}^{s}\\ \left|{\dddot{\theta }}^{s}(t)\right|\leq J_{\max}^{s} \end{array}\right.$$

where $V_{\max}^{s}$, $A_{\max}^{s}$, and $J_{\max}^{s}$ are the maximum velocity, maximum acceleration, and maximum pulse of the *s*th joint of the manipulator, respectively, and ${\dot{\theta }}^{s}(t)$, ${\ddot{\theta }}^{s}(t)$, and ${\dddot{\theta }}^{s}(t)$ are the actual velocity, acceleration, and pulse at time t of the *s*th joint of the manipulator, respectively.

In addition, during the spraying process of the robot, excessive torque input will cause the end of the manipulator to shake, resulting in low painting quality and easy damage to the body structure. Therefore, the dynamic constraints of each joint are limited as follows:

(38) $$\left|\tau^{s}(t)\right|\leq \tau_{\max}^{\;\;s}$$

where $\tau^{s}(t)$ is the torque of the *s*th joint of the manipulator at time t and $\tau_{\max}^{\;\;s}$ is the maximum torque of the joint.

### 4.3. Optimization Method Based on the NEDWOA

**Figure 3:**
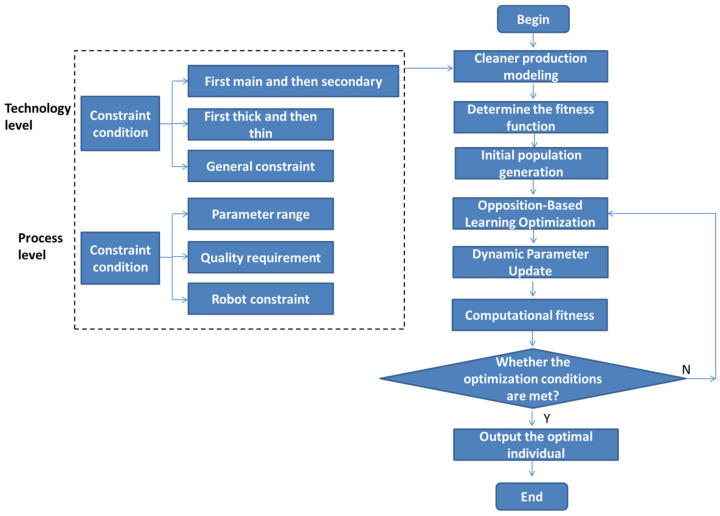
Process transformation flow chart based on NEDWOA.

The Analytic Hierarchy Process (AHP) and Criteria Importance Though Intercriteria Correlation (CRITIC) are combined with the weight, and the weight of fitness function is redistributed. The process transformation process based on the NEDWOA is shown in Figure 3.

#### Determination of Fitness Function

This paper uses the AHP method to construct the decision matrix for the consistency test and determine the subjective weight, *ω_s_*, to ensure the rationality of the optimization goal. The original experimental data were standardized, and the CRITIC method was used to determine the objective weight *ω_o_*:

(39) $$\omega_{o}=\frac{s_{j}c_{j}}{\sum_{j=1}^{n}s_{j}c_{j}},s_{j}=\sqrt{\frac{\sum_{j=1}^{n}{\left(\bar{y_{j}}-y_{ij}\right)}^{2}}{m-1}},c_{j}=\sum_{j=1}^{n}\left(1-r_{ij}\right)$$

where *s_j_* is the contrast strength, *c_j_* is conflict, *m* is the number of experimental groups, and *r_ij_* is the correlation coefficient between indicators.

The coefficient of variation, ζ, is used to calculate the subjective and objective combination weights:

(40) $$\omega_{j}=\zeta \omega_{o}+(1-\zeta )\omega_{s}=\frac{n}{n-1}G_{AHP}\omega_{o}+(1-\frac{n}{n-1}G_{AHP})\omega_{s}$$

where G_AHP_ is the weight coefficient of each index based on AHP.

The multi-objective optimization problem is transformed into a single-objective optimization problem by using the weight coefficient transformation method, and then the linear weighting calculation is carried out. For the objective function of the high-efficiency and low-consumption low-carbon model, the weights are determined according to the importance of the corresponding sub-objectives, and the fitness functions of the three sub-objective functions are expressed as:

(41) $$f=\left\{\begin{array}{c} \begin{array}{cc} \omega_{1}E_{P}+\omega_{2}C_{P}+\omega_{3}T_{P} & \;\mathrm{Process}\;\mathrm{level} \end{array}\\ \begin{array}{cc} \omega_{1}E_{T}+\omega_{2}C_{T}+\omega_{3}T_{T} & \mathrm{Technology}\;\mathrm{level} \end{array} \end{array}\right.$$

## 5. Case Study

**Figure 4:**
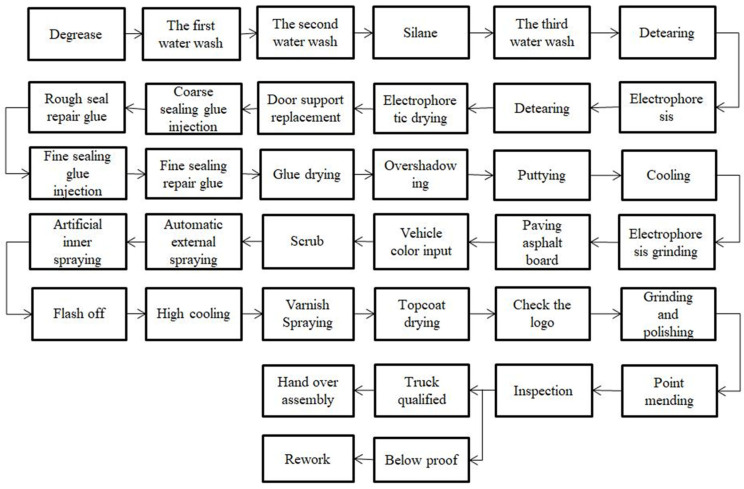
Process flow chart of body painting.

In order to verify the effectiveness of clean production in the spraying process based on the NEDWOA, this paper takes the TJ Company’s new energy vehicle body painting production system as an example to study. The painting workshop is responsible for the painting tasks relating to body, frame, cargo box, and plastic parts. The painting process is divided into two parts: the surface treatment technology of metal before painting and the construction technology of painting. The process flow diagram is shown in Figure 4. The main processes include pretreatment, electrophoresis drying, sealing glue injection, glue drying, electrophoresis grinding, spray painting, paint drying, and finishing.

### 5.1. Spraying Process Parameter Setting and Calculation

Among the spraying process parameters, the main parameters of rotary high-voltage electrostatic spraying, as an example, are spraying distance, spray cup speed, spraying flow, forming air, high-voltage electrostatic, rotary cup profiling speed, etc. The best spraying distance is set to 250 mm. The working speed of the rotating cup is 30,000 r/min during spraying, and the maximum no-load speed is 60,000 r/min. The working speed of the rotating cup varies according to the composition of the paint liquid. The rotation speed of the paint and the background paint in the solvent-based paint spraying is 30,000 r/min to 35,000 r/min (32,000 r/min is selected here) and 35,000 r/min to 45,000 r/min can be selected when spraying varnish (40,000 r/min is selected here). The thickness of the varnish coating film is 35 to 50 m, the thickness of the middle coating film is 30 to 45 m, and the thickness of the metal paint is 12 to 18 m [51]. The flash-drying time of the coating film was controlled at about 5 min. The wind speed of the circulating air filtration system that maintains the cleanliness of the spray room is generally controlled at 0.2~0.5 m/s. The number of coating overlaps was selected three times.

Assuming that the total amount of paint is not lost during the spraying process, and all of it is attached to the vehicle body, the spraying flow, Q, of each spin cup of the spraying robot is

(42) $$Q=\frac{S_{p}\delta }{TE\times NV\times N}$$

where Q is the spraying flow rate (mL/min), *S_p_* is the area needed to be sprayed (m^2^), *δ* is the dry film thickness (μm), *TE* is the coating efficiency of the coating, *NV* is the mass fraction (%) of paint solid under construction viscosity, and *N* is the number of robots in the station (the number of rotating cups).

The formed air is ejected from the annular gap at the rear side of the rotary cup to adjust the amplitude of the paint mist. The setting of forming air parameters is proportional to the paint flow rate and the rotating speed of the rotating cup. Generally, the parameter value of forming air is 100–350 NL/min.

The strength of the electrostatic field directly affects the electrostatic coating effect, such as coating efficiency, electrostatic effect, and the uniformity of the paint film. The suitable range of the average electric field intensity required for electrostatic coating is 3900–4000 V/cm. Electrostatic spraying stipulates that the safe distance between the electrode and the coated object is 250–300 mm. Different voltage settings should be adopted according to the different types of coatings sprayed. Intermediate coating is generally set as 65–75 kV. Varnish is set as 65–80 kV. Metallic paint is set as 50–65 kV. Due to the protruding corner of the body, it is easy to form the corner effect of electrostatic concentration. It is usually necessary to reduce the voltage setting, which can be controlled at 45–55 kV.

The spraying moving speed of the sprayer is related to the factors such as the spraying area completed on this station, the number of overlapping times during spraying, the production beat, the characteristics of the sprayer, and the use efficiency of the robot. The TCP speed, *v*, of the spraying robot can be calculated by the following Equation (35).

(43) $$v=\frac{S_{p}O_{f}}{Wt\eta_{R}K}$$

where S*_p_* is the spraying area of the automobile body (m^2^) and *O_f_* is the overlapping coefficient of spraying. When the overlapping area is 50%, *O_f_* = 2. When the overlapping area is 66%, *O_f_* = 3. When the overlapping area is 50%, *O_f_* = 4. *W* is the spray width, t is the spraying time, *η* is the use efficiency of the spraying robot, generally 90–95%, and K is the correction coefficient, generally 0.6–0.9.

According to the experimental conditions and the commonly recommended values of each factor, the values of each parameter are set in the experiment, as shown in Table 5.

### 5.2. Spraying Optimization Based on EDOWOA

Based on the AHP method, the subjective weight was determined and the judgment matrix was constructed. In this optimization process, considering the importance of the three (energy consumption, carbon emissions, and grinding time), the subjective weight values were 0.3333, 0.3333, and 0.3333. The process, robot feed speed, rotary cup speed, paint flow, and other parameters were changed to construct the data set. The original data were constructed and normalized, and the objective weight value was calculated by the CRITIC method. Among them, the calculated contrast strengths were 0.1306, 0.0537, and 0.1124 and the conflicts were 0.0463, 0.0621, and 0.0856, so the comprehensive objective weights were 0.3183, 0.1752, and 0.5064; therefore, the weights of each optimization objective of the whole spraying were 0.3258, 0.2543, and 0.4199.

**Figure 5:**
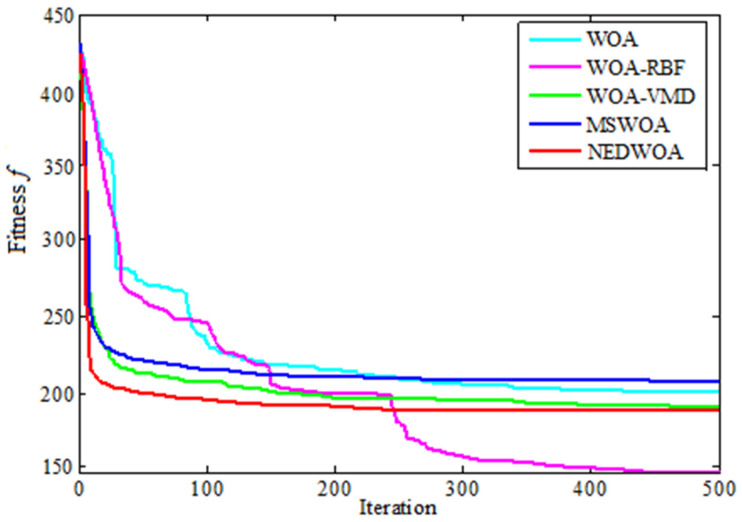
Multi-objective convergence diagram.

The algorithm program was written by software to randomly generate the population. A comparative analysis was conducted between the proposed NEDWOA algorithm and the existing methods (MSWOA, WOA-RBF, and WOA-VMD). The experimental parameters were consistent with the previous ones. After setting the parameters of the NED WOA, the fitness function was defined according to the objective function. Then, new individuals were generated by genetic operators. The algorithm convergence diagram of the spraying optimization process route is shown in Figure 5. It can be seen that the convergence speed of the NEDWOA is the fastest and more stable compared with other algorithms.

A comparative analysis was conducted between the proposed NEDWOA algorithm and the existing methods (WOA-RBF [27], WOA-VMD [28], and MSWOA [26]), under 250 iterations, with statistical evaluation of energy consumption, carbon emissions, and processing time per painted vehicle body, as shown in Table 6. The NEDWOA-optimized clean production framework demonstrated superior performance. This empirical validation confirms NEDWOA’s enhanced capability in balancing ecological and operational objectives, establishing it as a state-of-the-art solution for sustainable automotive painting systems.

**Figure 6:**
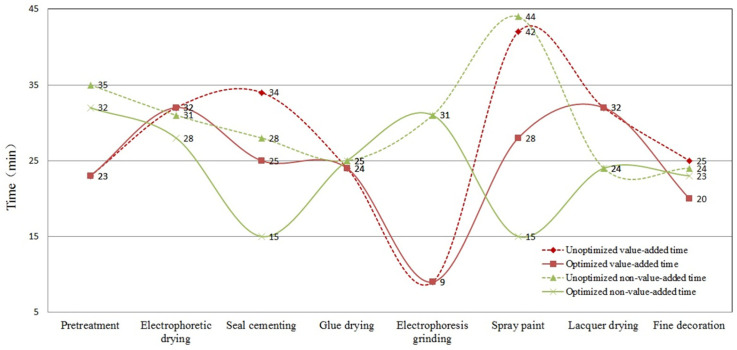
Comparison of value flow before and after body painting optimization.

The body painting workshop of TJ Automobile Manufacturing Co., Ltd. has a working time of six days/week. According to the order process and the basic data for body painting production control, the value flow of the body painting process before and after optimization is shown in Figure 6. It can be seen that good improvement results have been achieved.

According to the value stream map analysis, the value-added ratio, $VR_{b},$ before optimization and the value-added ratio, $VR_{a}$, after optimization are:

(44) $$VR_{b}=\frac{PT}{PT+NPT}=\frac{221}{221+274}=44.6\%$$

(45) $$R_{a}=\frac{PT}{PT+NPT}=\frac{193}{193+225}=46.2\%$$

where *PT* is the value-added operation time of the body painting production process, *PT* = $\sum_{i=1}^{n}T_{i}$, $T_{i}$ is the *i*th value-added operation time, *NPT* is the non-value-added operation time of the body painting production process, *NPT* = $\sum_{i=1}^{n}T_{i}^{\prime }$, and $T_{i}^{\prime }$ is the i th non-value-added operating time.

In addition, the optimized spraying clean production route has greatly improved the company’s production efficiency and product quality, as shown in Table 7.

## 6. Conclusions

This study establishes a clean production process model for automotive body painting from three perspectives: material flow, energy flow, and environmental emission flow. A multi-level, multi-objective optimization model is developed at both the process and operation layers, targeting painting energy consumption, painting carbon emissions, and painting time. An improved whale optimization algorithm (NEDWOA) based on AHP–CRITIC combined weighting is proposed to solve the optimization problem. Taking the body coating process of TJ company as an example, the experimental results show that the production efficiency of the optimized process route is increased by 42.1%, the exhaust gas purification rate is 98%, the manufacturing cost is reduced by 17.9%, and energy consumption is reduced by 18.2%. These results significantly enhance production profitability and quality, providing management and practical experience for domestic automotive manufacturers in clean production implementation. Additionally, the methodology offers strategic insights for advancing sustainable practices in other industries.

## Figures and Tables

**Table 1:** Research Status of whale optimization algorithm.

Improvement Form	Specific Algorithm	Improve Features
Integrate with other algorithms	PSO-WOA [21]	PSO is used for the development phase and WOA is used for the exploration phase.
	MDE-WOA [22]	It effectively improves the problem of local optimization and reduces the complexity.
	WOA-PS [23]	Combining with the pattern search algorithm for power system planning.
	ESSAWOA [24]	The nonlinear parameter SSA is used in the development stage to accelerate the convergence speed of the development stage.
	WOA-Salp [25]	Combining with the Salp group algorithm to improved search optimization capabilities.
	HS-WOA [26]	The global search ability and convergence performance of the algorithm are enhanced by combining multiple strategies.
	WOA-FPA [27]	Combining with Flower Pollination Algorithm to solve complex nonlinear systems and unconstrained optimization problems.
Strategic innovation	IWPA [28]	The exploration ability of whale optimization algorithm is improved.
	IWOA [29]	Perform bearing fault diagnosis.
	EWOA [30]	An enhancement of the meta-heuristic whale optimization algorithm using for maximum power point tracking of variable speed wind turbine.
	RLWOA [31]	Refraction learning-based whale optimization algorithm. Performance tests were performed on 1,001,000, and 10,000 dimensions of the benchmark function.
	Improved whale optimization algorithm based on inertial weight [32]	Inertia weight is introduced to improve the convergence performance.
	MWOA [33]	Population initialization optimization, position update mechanism improvement, convergence factor optimization.
	WOALam [34]	WOA based on Lamarckian learning can balance global and local search capabilities well.
Multi-strategy integration	HWOA [35]	The adaptive strategy is introduced to update the whale population position by using the local search mechanism of bat algorithm, and then improve the global optimization and local optimization of the balance algorithm by using the adaptive strategy.
	MWOA [36]	A modified whale optimization algorithm to overcome delayed convergence in artificial neural networks.
	AWOA [37]	The adaptive parameter adjustment strategy is introduced, which has better competitive performance compared with the standard WOA.
	WAROA [38]	Military combat strategy is introduced to enhance the population collaborative search ability and global optimization performance.
	MSWOA [39]	The initial population data of chaotic initialization strategy are introduced, the Levy flight strategy is introduced to balance local search and global search, and the improved position correction mechanism is used to further improve the exploration level.
	HWOAG [40]	The information collection mechanism is introduced to enhance the knowledge accumulation ability of the algorithm.
	CQAWOA [41]	The chaotic mapping initialization strategy and the quadratic adversarial learning strategy are introduced to construct a negative feedback adjustment network in the algorithm
	WOA-CNN-BiLSTM [42]	An improved bidirectional long short-term memory network method combining convolutional neural networks and whale optimization algorithm was proposed.
	GWO–WOA [43]	A novel hybrid deep learning optimization technique for human activity recognition.

**Table 2:** Information about benchmark functions.

Name	Equation	Dimension	Search Space
Sphere	$F_{1}(x)=\sum_{k=1}^{D}x_{k}^{2}$	30	[−100, 100]
Schwefel 2.22	$F_{2}(x)=\sum_{k=1}^{D}\left|x_{k}\right|+\prod_{k=1}^{D}\left|x_{k}\right|$	30	[−10, 10]
Schwefel 1.12	$F_{3}(x)={\sum_{k=1}^{D}\left(\sum_{l=1}^{k}x_{l}\right)}^{2}$	30	[−100, 100]
Schwefel 2.21	$F_{4}(x)=\underset{k}{\max}\left[\left|x_{k}\right|,1\leq k\leq D\right]$	30	[−100, 100]
Rosenbrock	$F_{5}(x)=\sum_{k=1}^{D-1}\left[100{\left((x_{k+1}-x_{k}^{2})\right)}^{2}+{\left(x_{k}-1\right)}^{2}\right]$	30	[−30, 30]
Step	$F_{6}(x)=\sum_{k=1}^{D}{\left(\left|x_{k}+0.5\right|\right)}^{2}$	30	[−100, 100]
Quartic	$F_{7}(x)=\sum_{k=1}^{D}kx_{k}^{4}+random(0,1)$	30	[−1.28, 1.28]
Schwefel 2.26	$F_{8}(x)=\sum_{k=1}^{D}-x_{k}\sin(\sqrt{\left|x_{k}\right|})$	30	[−500, 500]
Rastrigin	$F_{9}(x)=\sum_{k=1}^{D}\left[x_{k}^{2}-10\cos(2\pi x)+10\right]$	30	[−5.12, 5.12]
Ackley	$\begin{array}{cc} F_{10}(x) & =-20\exp(-0.2\sqrt{\frac{1}{D}\sum_{k=1}^{D}x_{k}^{2}}-\exp(\frac{1}{D}\sum_{k=1}^{D}\cos2\pi x_{k}))\\ & +20+e \end{array}$	30	[−32, 32]
Alpine	$F_{11}(x)=\sum_{k=1}^{D}\left|x_{k}\sin(x_{k})+0.1x_{k}\right|$	30	[−10, 10]
Penalized 1.1	$\begin{array}{cc} F_{12}(x) & =\frac{\pi }{D}\cdot (10\sin(\pi y_{1})+{\left(y_{k}-1\right)}^{2})+\\ & \frac{\pi }{D}\cdot \sum_{k=1}^{D-1}{\left(y_{k}-1\right)}^{2}[1+10\sin^{2}(\pi y_{k+1})]\\ & +\sum_{k=1}^{D}\mu (x_{k},10,100,4)\\ y_{k} & =1+\frac{x_{k}+1}{4},\mu (x_{k},p,a,m)=\left\{\begin{array}{l} p{\left(x_{k}-a\right)}^{m},x_{k}>a\\ 0,-a<x_{k}<a\\ p{\left(-x_{k}-a\right)}^{m},x_{k}<-a \end{array}\right. \end{array}$	30	[−50, 50]
Penalized 1.2	$\begin{array}{c} F_{13}(x)=0.1\left\{\sin^{2}(3\pi x_{1})+\sum_{k=1}^{D}{\left(x_{k}-1\right)}^{2}[1+\sin^{2}(3\pi x_{k}+1)]\right.+\\ \left.{\left(x_{k}-1\right)}^{2}[1+\sin^{2}(2\pi x_{k})]+\sum_{k=1}^{D}\mu (x_{k},5,100,4)\right\} \end{array}$ $\mu (x_{k},p,a,m)=\left\{\begin{array}{l} p{\left(x_{k}-a\right)}^{m},x_{k}>a\\ 0,-a<x_{k}<a\\ p{\left(-x_{k}-a\right)}^{m},x_{k}<-a \end{array}\right.$	30	[−50, 50]
Michalewicz	$F_{14}(x)={\left(\frac{1}{500}+\sum_{l=1}^{25}\frac{1}{l+\sum_{k=1}^{D}{\left(x_{k}-a_{kl}\right)}^{6}}\right)}^{-1}$	2	[−65.536, 65.536]
Kowalik	$F_{15}(x)={\sum_{k=1}^{11}\left[a_{i}-\frac{x_{1}(b_{k}^{2}+b_{k}x_{2})}{b_{k}^{2}+b_{k}x_{3}+x_{4}}\right]}^{2}$	4	[−5, 5]
Six-Hump Camel-Back	$F_{16}(x)=4x_{1}^{2}-2.1x_{1}^{4}+\frac{1}{3}x_{1}^{6}+x_{1}x_{2}-4x_{2}^{2}+4x_{2}^{4}$	2	[−5, 5]
Branin	$F_{17}(x)={\left(x_{2}-\frac{5.1}{4\pi^{2}}x_{1}^{2}+\frac{5}{\pi }x_{1}-6\right)}^{2}+10(1-\frac{1}{8\pi })\cos x_{1}+10$	2	[−5, 5]
Goldstein-Price	$\begin{array}{l} F_{18}(x)=[1+{\left(x_{1}+x_{2}+1\right)}^{2}(19-14x_{1}+3x_{1}^{2}-14x_{2}+6x_{1}x_{2}+3x_{2}^{2})]\\ \times [30+{\left(2x_{1}-3x_{2}\right)}^{2}\times (18-32x_{1}+12x_{1}^{2}+48x_{2}-36x_{1}x_{2}+27x_{2}^{2})] \end{array}$	2	[−2, 2]
Hartmann-3D	$F_{19}(x)=-\sum_{k=1}^{4}c_{k}\exp(-\sum_{l=1}^{3}a_{kl}{\left(x_{l}-p_{kl}\right)}^{2})$	3	[0, 1]
Hartmann-6D	$F_{20}(x)=-\sum_{k=1}^{4}c_{k}\exp(-\sum_{l=1}^{6}a_{kl}{\left(x_{l}-p_{kl}\right)}^{2})$	6	[0, 1]
Shekel 5	$F_{21}(x)=-\sum_{k=1}^{5}{\left[(X-a_{k}){\left(X-a_{k}\right)}^{T}c_{k}\right]}^{-1}$	4	[0, 10]
Shekel 7	$F_{22}(x)=-\sum_{k=1}^{7}{\left[(X-a_{k}){\left(X-a_{k}\right)}^{T}c_{k}\right]}^{-1}$	4	[0, 10]
Shekel 10	$F_{23}(x)=-\sum_{k=1}^{10}{\left[(X-a_{k}){\left(X-a_{k}\right)}^{T}c_{k}\right]}^{-1}$	4	[0, 10]

**Table 3:** Effect of each proposed component.

Functions	Evaluation Indexes	WOAa	WOAo	WOAσ
F1	Avg	4.52 × 10^−89^	6.90 × 10^−62^	2.74 × 10^−114^
	Std	2.73 × 10^−88^	3.26 × 10^−64^	3.66 × 10^−117^
F2	Avg	6.38 × 10^−50^	7.39 × 10^−34^	4.42 × 10^−76^
	Std	5.71 × 10^−49^	3.82 × 10^−33^	2.55 × 10^−78^
F3	Avg	2.74 × 10^−7^	7.16 × 10^−9^	3.41 × 10^−13^
	Std	2.89 × 10^−6^	2.07 × 10^−8^	3.78 × 10^−14^
F4	Avg	5.58 × 10^−38^	7.19 × 10^−33^	3.52 × 10^−60^
	Std	6.59 × 10^−39^	2.44 × 10^−34^	4.58 × 10^−59^
F5	Avg	3.06 × 10^0^	2.76 × 10^1^	3.74 × 10^−1^
	Std	3.02 × 10^−1^	4.73 × 10^−1^	1.62 × 10^−1^
F6	Avg	6.25 × 10^−4^	5.51 × 10^−5^	4.66 × 10^−6^
	Std	3.27 × 10^−5^	1.90 × 10^−6^	5.42 × 10^−7^
F7	Avg	2.78 × 10^−3^	1.13 × 10^−4^	3.51 × 10^−5^
	Std	8.52 × 10^−4^	2.71 × 10^−5^	2.46 × 10^−5^
F8	Avg	−1.22 × 10^4^	−1.22 × 10^4^	−1.24 × 10^4^
	Std	1.41 × 10^2^	6.97 × 10^2^	3.22 × 10^1^
F9	Avg	2.54 × 10^−35^	1.89 × 10^−35^	7.22 × 10^−61^
	Std	4.37 × 10^−35^	1.04 × 10^−34^	7.25 × 10^−63^
F10	Avg	3.25 × 10^−15^	2.31 × 10^−15^	4.72 × 10^−17^
	Std	1.77 × 10^−15^	1.77 × 10^−15^	5.66 × 10^−18^
F11	Avg	3.47 × 10^−9^	3.47 × 10^−9^	8.56 × 10^−39^
	Std	2.64 × 10^−11^	2.64 × 10^−11^	4.77 × 10^−38^
F12	Avg	2.90 × 10^−3^	2.90 × 10^−3^	6.35 × 10^−4^
	Std	1.33 × 10^−2^	1.33 × 10^−2^	5.21 × 10^−5^
F13	Avg	7.61 × 10^−4^	7.61 × 10^−4^	5.26 × 10^−11^
	Std	2.56 × 10^−4^	2.56 × 10^−4^	3.42 × 10^−10^
F14	Avg	2.44 × 10^0^	2.44 × 10^0^	1.89 × 10^0^
	Std	2.50 × 10^0^	2.50 × 10^0^	1.24 × 10^−1^
F15	Avg	5.42 × 10^−4^	5.42 × 10^−4^	3.98 × 10^−4^
	Std	4.33 × 10^−4^	4.33 × 10^−4^	5.26 × 10^−6^
F16	Avg	−1.03 × 10^0^	−1.03 × 10^0^	−1.03 × 10^0^
	Std	5.55 × 10^−6^	5.55 × 10^−6^	3.56 × 10^−7^
F17	Avg	3.98 × 10^−1^	3.98 × 10^−1^	3.98 × 10^−1^
	Std	6.54 × 10^−4^	6.54 × 10^−4^	6.42 × 10^−5^
F18	Avg	3.00 × 10^0^	3.00 × 10^0^	3.00 × 10^0^
	Std	6.71 × 10^−4^	6.71 × 10^−4^	2.53 × 10^−4^
F19	Avg	−3.86 × 10^0^	−3.86 × 10^0^	−3.86 × 10^0^
	Std	8.16 × 10^−5^	8.16 × 10^−5^	3.74 × 10^−6^
F20	Avg	−3.25 × 10^0^	−3.25 × 10^0^	−3.29 × 10^0^
	Std	9.03 × 10^−2^	9.03 × 10^−2^	7.69 × 10^−2^
F21	Avg	−1.01 × 10^1^	−1.01 × 10^1^	−1.02 × 10^1^
	Std	5.48 × 10^−1^	5.48 × 10^−1^	8.76 × 10^−2^
F22	Avg	−1.04 × 10^1^	−1.04 × 10^1^	−1.04 × 10^1^
	Std	5.44 × 10^−3^	5.44 × 10^−3^	1.68 × 10^−3^
F23	Avg	−1.04 × 10^1^	−1.04 × 10^1^	−1.04 × 10^1^
	Std	5.11 × 10^−4^	5.11 × 10^−4^	5.86 × 10^−6^

**Table 4:** Comparison of optimization results for related algorithms.

Functions	Evaluation Indexes	WOA	WOA-RBF	WOA-VMD	MSWOA	NEDWOA
F_1_	Avg	7.91 × 10^−74^	8.32 × 10^−93^	**0.00 × 10^0^**	3.58 × 10^−89^	**0.00 × 10^0^**
	Std	4.32 × 10^−74^	1.23 × 10^−64^	**0.00 × 10^0^**	2.40 × 10^−84^	**0.00 × 10^0^**
F_2_	Avg	1.86 × 10^−49^	7.59 × 10^−72^	5.23 × 10^−52^	5.27 × 10^−197^	**0.00 × 10^0^**
	Std	2.39 × 10^−48^	2.56 × 10^−93^	3.43 × 10^−51^	3.21 × 10^−198^	**0.00 × 10^0^**
F_3_	Avg	4.31 × 10^−6^	5.43 × 10^1^	**0.00 × 10^0^**	4.39 × 10^−41^	**0.00 × 10^0^**
	Std	2.93 × 10^−5^	4.67 × 10^2^	**0.00 × 10^0^**	2.41 × 10^−40^	**0.00 × 10^0^**
F_4_	Avg	7.25 × 10^−14^	2.07 × 10^−4^	**3.16 × 10** **^−^** **^187^**	1.51 × 10^−27^	1.35 × 10^−93^
	Std	3.97 × 10^−12^	1.40 × 10^−4^	**2.78 × 10^−187^**	6.84 × 10^−27^	2.57 × 10^−95^
F_5_	Avg	2.79 × 10^1^	**2.06 × 10** **^−^** **^2^**	1.86 × 10^−1^	2.62 × 10^1^	2.94 × 10^−1^
	Std	7.63 × 10^−1^	**2.23 × 10** **^−^** **^2^**	3.75 × 10^−1^	3.49 × 10^0^	3.39 × 10^−1^
F_6_	Avg	7.79 × 10^−3^	2.34 × 10^−2^	6.63 × 10^−1^	1.53 × 10^−4^	**5.21 × 10^−9^**
	Std	5.32 × 10^−2^	2.51 × 10^−1^	4.27 × 10^−1^	1.89 × 10^−3^	**1.46 × 10^−8^**
F_7_	Avg	5.51 × 10^−3^	7.25 × 10^−4^	4.76 × 10^−4^	**3.28 × 10** **^−^** **^6^**	7.45 × 10^−5^
	Std	1.15 × 10^−2^	3.85 × 10^−4^	7.46 × 10^−4^	**3.21 × 10** **^−^** **^5^**	6.96 × 10^−5^
F_8_	Avg	−8.98 × 10^3^	−10,745.9	−12,569.52	−12,621.32	−**12,568.81**
	Std	1.91 × 10^3^	1.76 × 10^3^	3.36 × 10^−2^	2.57 × 10^−2^	**1.21 × 10^−2^**
F_9_	Avg	1.06 × 10^−21^	7.28 × 10^−5^	**0.00 × 10^0^**	**0.00 × 10^0^**	**0.00 × 10^0^**
	Std	2.39 × 10^−21^	7.35 × 10^−5^	**0.00 × 10^0^**	**0.00 × 10^0^**	**0.00 × 10^0^**
F_10_	Avg	5.39 × 10^−15^	6.39 × 10^−3^	4.47 × 10^−16^	4.56 × 10^−16^	**5.76 × 10^−18^**
	Std	2.93 × 10^−6^	3.71 × 10^−3^	5.58 × 10^−15^	2.32 × 10^−14^	**6.56 × 10^−21^**
F_11_	Avg	1.26 × 10^−2^	1.38 × 10^0^	**3.96 × 10** **^−^** **^200^**	9.37 × 10^−75^	1.26 × 10^−45^
	Std	3.97 × 10^−1^	5.21 × 10^0^	**0.00 × 10^0^**	3.96 × 10^−74^	2.87 × 10^−46^
F_12_	Avg	3.05 × 10^−3^	**6.41 × 10^−6^**	7.27 × 10^−3^	9.32 × 10^−4^	6.85 × 10^−5^
	Std	7.60 × 10^−2^	**6.39 × 10^−6^**	6.47 × 10^−3^	2.89 × 10^−3^	3.43 × 10^−5^
F_13_	Avg	8.97 × 10^0^	7.57 × 10^−5^	9.81 × 10^−3^	1.41 × 10^−2^	**7.15 × 10^−12^**
	Std	6.69 × 10^0^	6.56 × 10^−5^	1.51 × 10^−2^	1.74 × 10^−2^	**1.78 × 10^−11^**
F_14_	Avg	3.76 × 10^0^	−1.5037	−**1.0246**	−1.5496	1.24 × 10^0^
	Std	2.59 × 10^0^	2.45 × 10^−1^	**6.32 × 10^−2^**	2.81 × 10^−1^	1.38 × 10^−1^
F_15_	Avg	1.81 × 10^−3^	8.79 × 10^−4^	4.51 × 10^−3^	5.13 × 10^−4^	**3.79 × 10^−4^**
	Std	2.39 × 10^−3^	5.91 × 10^−4^	7.49 × 10^−3^	2.19 × 10^−4^	**3.94 × 10^−4^**
F_16_	Avg	−0.99997	−**1.0316**	−**1.0316**	−**1.0316**	−**1.0316**
	Std	2.93 × 10^−5^	3.46 × 10^−6^	1.61 × 10^−5^	2.78 × 10^−5^	**1.19 × 10^−7^**
F_17_	Avg	0.42718	**0.3980**	0.3991	**0.3980**	0.39996
	Std	3.97 × 10^−1^	**8.31 × 10^−6^**	2.71 × 10^−4^	3.52 × 10^−5^	2.78 × 10^−5^
F_18_	Avg	3.001	3.0022	4.3658	1.15 × 10^1^	**3.00007**
	Std	7.62 × 10^−1^	2.23 × 10^−4^	7.5019 × 10^0^	1.30 × 10^1^	**1.78 × 10^−4^**
F_19_	Avg	−3.8349	−3.8467	−3.7589	−3.84	−**3.8563**
	Std	5.92 × 10^−2^	5.37 × 10^−2^	1.89 × 10^−1^	4.79 × 10^−2^	**3.66 × 10^−7^**
F_20_	Avg	−3.1533	−3.2054	−2.9182	−3.1220	−**3.2723**
	Std	1.18 × 10^−1^	9.35 × 10^−2^	0.2876	0.1336	**6.49 × 10^−2^**
F_21_	Avg	−8.2895	−10.1443	−10.0391	−10.0279	−**10.1526**
	Std	2.73 × 10^0^	7.54 × 10^−3^	1.30 × 10^−1^	2.16 × 10^−1^	**2.23 × 10^−3^**
F_22_	Avg	−8.7296	−10.2382	−10.2612	−10.2975	−**10.4023**
	Std	3.42 × 10^0^	1.05 × 10^0^	2.04 × 10^−1^	1.18 × 10^−1^	**9.11 × 10^−3^**
F_23_	Avg	−7.1025	−10.3469	−10.3186	−10.3227	−**10.3732**
	Std	2.37 × 10^0^	7.56 × 10^−2^	1.22 × 10^−1^	8.53 × 10^−2^	**5.13 × 10^−2^**
*p*/*h*		1.97 × 10^−7^/+	4.16 × 10^−6^/+	5.16 × 10^−6^/+	1.47 × 10^−5^/+	
Total rank		5	4	3	2	**1**

**Table 5:** Experimental parameter settings.

Parameter	Taking Values	Parameter	Taking Values
spraying distance	250 mm	static voltage U	60 kV
cup speed n	priming paint (32 kr/min)	average film thickness δ	priming paint (20 μm)
	inter coatings (32 kr/min)		inter coatings (40 μm)
	final coating (40 kr/min)		varnish (40 μm)
			metallic paint (16 μm)
paint flux Q	300 cc/min	forming air flow rate	300 NL/min
travel speed v	400 mm/s	plane tilt angle α	0°

**Table 6:** Performance comparison between different algorithms.

Metric	MSWOA	WOA-RBF	WOA-VMD	NEDWOA
Energy Consumption (kWh)	352	367	358	**346**
Carbon Emissions (kg CO_2_)	251	263	259	**245**
Processing Time (h)	4.9	5.8	6.1	**4.2**
Total consumption	180.57	188.88	185.06	**176.79**

**Table 7:** TJ company body painting production process transformation effect.

Serial Number	Improvement Goals	Original Production Data	Current Production Data	Analysis Results
1	Production efficiency improvement	675/month	**959/month**	42.1%
2	Increased value-added ratio of production	44.6%	**46.2%**	1.6%
3	Production personnel reduction rate	16 patients	**12 patients**	25%
4	Production cost reduction	719 yuan/piece	**590 yuan/piece**	17.9%
5	Improvement of production quality	defective index 15%	**defective index 7%**	8%
6	Exhaust gas purification rate	32%	**98%**	66%
7	Energy consumption	423 kwh	**346 kwh**	18.2%

## Data Availability

The data that support the findings of this study are available within the paper.
